# Children's Food and Drink Purchasing Behaviour “Beyond the School Gate”: The Development of a Survey Module

**DOI:** 10.5402/2013/501450

**Published:** 2013-02-14

**Authors:** Wendy J. Wills, Jennie I. Macdiarmid, Lindsey F. Masson, Catherine Bromley, Leone Craig, Geraldine McNeill

**Affiliations:** ^1^Centre for Research in Primary and Community Care, University of Hertfordshire, College Lane, Hatfield AL10 9AB, UK; ^2^Rowett Institute of Nutrition and Health, University of Aberdeen, Polwarth Building, Foresterhill, Aberdeen AB25 2ZD, UK; ^3^Institute of Applied Health Sciences, University of Aberdeen, Polwarth Building, Foresterhill, Aberdeen AB25 2ZD, UK; ^4^Centre for Population Health Sciences, The University of Edinburgh Medical School, Teviot Place, Edinburgh EH8 9AG, UK; ^5^Public Health Nutrition Research Group, University of Aberdeen, Polwarth Building, Foresterhill, Aberdeen AB25 2ZD, UK

## Abstract

Many children eat a diet which supplies a higher than recommended amount of nonmilk extrinsic sugars and saturated fatty acids. The school setting is often targeted for nutrition intervention as many children consume food at school. In Scotland, attempts have been made to improve the nutritional content of food in schools and attention has now turned to food and drink available “beyond the school gate.” This paper describes the development of a module on food and drink purchasing behaviour. The Food Purchasing Module was designed to collect data, for the first time, from a representative sample of children aged 8–16 years about food and drinks purchased on the way to/from school, during break time/free periods, and at lunchtime, from outlets around schools. Cognitive testing of the module highlighted that younger children find self-completion questionnaires problematic. Older children have fewer problems with self-completion questionnaires but many do not follow question routing, which has implications for the delivery of future surveys. Development of this survey module adds much needed evidence about effectively involving children in surveys. Further research exploring food and drinks purchased beyond the school gate is needed to continue to improve the nutritional quality of children's diets.

## 1. Introduction

Many children's intakes of nonmilk extrinsic sugars (NMESs) and saturated fatty acids (SFA) fail to meet dietary targets for these nutrients [1]. Diets high in NMES and SFA are likely to contribute to the continued high prevalence of obesity and overweight amongst children. The Scottish Government has taken several steps to try to improve children's diets, including interventions in the school setting. The Schools (Health Promotion and Nutrition) Scotland Act [2], passed in 2007, sets out nutritional standards for school meals and prohibits the sale of foods high in sugar in primary and secondary schools (e.g., confectionery and sweetened soft drinks) and limits the sale of foods high in fat (e.g., fried foods and most snacks). Once the act was implemented across all schools in Scotland, attention turned to some of the other factors which could be influencing school-aged children's diets. One suggestion was to address children's purchasing of food and drinks high in fat and/or sugar in outlets found in the vicinity of schools. Such outlets are referred to in Scotland's Obesity Route Map Action Plan as being “beyond the school gate” [3]. 

Evidence suggests that the presence and geographical density of food outlets (this includes restaurants and stores) influences the diet of local people, including children and adolescents, thereby contributing to the so-called obesogenic environment [4, 5], though whether that influence is positive or negative, in terms of impacting on rates of obesity and diet quality, is uncertain [6, 7]. It is clear, however, that the local food environment is associated with socioeconomic inequalities in diet, obesity, and health [7], though the evidence is mixed regarding an association between deprivation and the prevalence of fast food outlets [8, 9]. 

There is little evidence in Scotland of the extent to which children access food and drink in outlets beyond the school gate, though one study involving older children (aged 14–16 years) at three schools in Scotland reported that over half of young people left the school grounds every day to purchase food and that factors associated with “personal liberty” (e.g., being treated as an adult at local stores and a desire to make a personal choice about where to eat) influenced decisions to purchase food beyond the school gate [10]. Another study found that some children wanted to escape the school environment at lunchtime [11]. 

In England, an in-depth study of two secondary schools found that food bought in shops close to schools contributed 23% to children's energy intakes, a quarter of which came from total sugars (15% from NMES) [12]. Of the children given permission by schools to leave the grounds at lunchtime, at least 97% purchased food outside school [12]. Many schools in the UK do not allow children to leave the school grounds during the school day, particularly younger children, which could act as a barrier to purchases beyond the school gate, at least for those who obey the rules. However, many secondary school canteens are not big enough to seat all their students and canteens are often reported by young people to be noisy, unpleasant, and offering a poor choice of food [7–9]; therefore, such factors can act as “pushes” for children to seek food and drink elsewhere, regardless of the rules about leaving the school grounds. 

Children can also purchase food and drink on the way to or from school (and during break times and free periods); therefore, lunchtime is not the only opportunity for purchases to be made [12]. As there are reported to be an average of 24 food outlets around secondary schools in one large city in Scotland [8], there is certainly opportunity for local outlets to attract young consumers with price promotions and a fast service, both of which are thought to be important factors within the local food environment [7]. 

The limited evidence from within the UK and Scotland more specifically and the recent policy spotlight on food and drink purchasing beyond the school gate informed the decision to develop a new survey module on food purchasing behaviour, as part of the 2010 Survey of Diet among Children in Scotland [13]. This was the first attempt to obtain data from a representative sample of children in Scotland about their food and drink purchasing habits beyond the school gate and was therefore an important first step towards considering whether such purchases influence dietary intakes of NMES and SFA. The overall aim of the food purchasing module (FPM) was to assess the food and drink purchasing habits outside of school on school days among primary (aged 8–11 years) and secondary (aged 11–16 years) school children living in Scotland. The specific objectives were the following.

- To assess the opportunities for children to purchase food and drink outside of school.
- To estimate the proportion of children purchasing food and drink outside of school.
- To identify the types of foods and drinks children are purchasing outside of school.
- To explore some of the factors that influence whether children go outside of school to purchase food or drink.

The aim of this paper is to describe the development of the food purchasing module (FPM). 

## 2. Method

### 2.1. Design of the Food Purchasing Module (FPM)

The FPM was designed to be administered as part of the 2010 Survey of Diet among Children in Scotland [13]. The dietary survey aimed to monitor dietary trends of children in Scotland and was similar to the design of the earlier 2006 Survey of Sugar Intake Among Children in Scotland [1], which entailed parents and children completing a Food Frequency Questionnaire (FFQ) before taking part in a face-to-face Computer Assisted Personal Interview (CAPI) with an interviewer. Several decisions had to be made about the design of the new FPM, including whether to include all school-aged children in the sample, how to deliver the FPM to children alongside the dietary survey, which time points around the school day to ask children about, and how best to design questions which would address the study's objectives.

Whilst the main dietary survey included children aged 3–16 years, the FPM was concerned with food and drink purchased by children for themselves during the school day; therefore, children aged 3-4 years were automatically excluded as they had not yet reached school age. It was felt that most children aged 5–7 years would be unlikely to purchase food *for themselves* therefore they were also excluded. The FPM was designed to be administered to children aged 8–16 years. 

It was considered that some children may not wish to discuss their food and drink purchasing behaviours in front of their parents; therefore, some questions were designed to be delivered through a self-completion paper questionnaire (SCQ) and other less sensitive questions would be delivered through the CAPI of the main dietary survey. The CAPI was designed so that parents of primary school children (aged 8–11 years) would answer the questions, with some input from the child, whilst secondary school children aged 11–16 years would complete the CAPI themselves with input from their parent(s) as needed. The SCQ was initially designed for completion by all children aged 8–16 years. 

The authors considered that children are likely to display different behaviours with regard to food and drinks purchased at different points during the school day; therefore, questions were designed to assess purchasing behaviour at four time points: (i) on the way to school, (ii) at break time and during free periods, (iii) lunchtime, and (iv) after leaving school. Whilst the rules about secondary school children leaving school during break and lunchtime are variable across schools in Scotland, it was felt that very few primary school children would be allowed to leave school during break or lunchtime; therefore, children aged 8–11 years were asked questions about food purchasing on the way to/from school only. The survey concentrated on all 4 time points for secondary school children aged 11–16 years.

In order to address the FPM objectives, it was important to consider and define what constituted “opportunities” for purchasing food and drink on the way to/from school and it was agreed that this meant “the child walking or cycling past places selling food or drinks.” It was felt that going past places selling food or drinks in a car, bus, taxi, or other vehicles did not represent an opportunity for children to purchase anything, without requesting the adult driving the vehicle to stop. At break time/free periods and lunchtime, the opportunity to purchase food or drinks was defined as “the child being able to get to places outside of school that sell food or drinks.” Opportunities to purchase were based on parents' and children's perceptions of the type of places they considered accessible and whether they perceived that these outlets sell food or drinks. The FPM made it clear that we were interested in food and drinks children purchased themselves; this was designed to exclude purchases made for them by adults or other children. It is worth noting that the FPM was concerned with the *purchasing *of food and drinks; therefore, this might not relate to *consumption* as we cannot determine if the food and drinks purchased were actually consumed. 

When deciding which factors to explore in relation to reasons for children purchasing food and drinks outside of school, we drew on the authors' expertise and other relevant research and literature [10–12, 14]. The factors asked about in relation to buying food/drink on the way to/from school included purchasing food because of hunger/thirst, copying friends, not having anything to eat/drink from/at home, and wanting food/drink to eat later. In relation to leaving school at break/free periods or lunchtime questions were asked about wanting a break from school, preferring the food sold outside school, and exercising choice about where to purchase food. Questions were also asked about why children did *not *leave school at break and lunchtime including, not having enough money, having nowhere to go, not being hungry/thirsty, taking food/drink to school from home, not having time to purchase anything, and preferring to purchase food/drink at school. Children were also given the opportunity to specify an “other” reason, although very few did so.

Whilst the questions exploring the reasons for purchasing/not purchasing food and drinks are not exhaustive, they provide baseline information from a representative sample for the first time and can be developed further in future surveys. 

The main study received ethics approval from the Nat Cen Social Research Ethics Committee; the cognitive testing phase received ethics approval from the University of Aberdeen's College Ethics Review Board.

### 2.2. Cognitive Testing of the FPM

As this was a new survey module, cognitive testing was carried out to check if children understood the wording and routing of the questions and could navigate the self-completion questionnaire [15]. Parents' understanding of the CAPI questions was also tested. Seventeen interviews were conducted with primary and secondary school children, and five interviews were conducted with parents who had primary school-aged children.

During the cognitive interviews, a combination of “think-aloud” and probing techniques were used. Children and parents were asked to describe what they were thinking as they tried to navigate the SCQ or answer the CAPI questions. Observations were recorded about the way each child or parent worked their way through the module questions (e.g., whether they read the instructions for each section). Children completed the SCQ without help from the interviewer, to replicate the main stage of the survey, and, similarly, the CAPI was administered exactly as it would be administered in the main phase of the survey. The interviewer also probed participants about their understanding of some of the terms used in the questions, to determine if children and parents understood them in the way intended.

The cognitive interviews highlighted that primary and secondary school children found navigating their way through the SCQ problematic and that primary school children struggled to complete the SCQ. However, there were few problems with the interview-based (CAPI) questions as they were originally designed.

#### 2.2.1. Primary School Children

Nearly all the primary school children had problems navigating through the SCQ and could not follow the routing of the questions (e.g., being asked to “go to question 6” if they gave a negative response to an earlier question); this caused the majority of their problems with the SCQ. Only one child followed the “go to next question…” instructions. Some of the youngest children were not able to read words in some of the questions or did not know what the words meant. Some did not understand the instructions at the start of the questionnaire while others did not read the instructions at all. Some children thought the instructions were a question and very few read the “tick one box” instruction. Questions asking about the type of food or drinks purchased outside of school created a lot of confusion among the primary school children as they were unsure what food this related to in their day.

Children's interpretation of some of the terms used to describe foods was probed and in general their understanding was good. The term “packets of snacks” (which referred to savoury snacks) was interpreted by most children to mean sweet snacks. Most children were familiar with the terms “diet” and “non-diet” drinks but many were less familiar with the meaning of “low sugar drinks.”

#### 2.2.2. Secondary School Children

The majority of secondary school children did not read the instructions at the start of the questionnaire and went straight to the first question. Most of them had some problems navigating through the questionnaire following the routing of the questions. Most of them did not read or follow the “go to…” instructions.

The majority of questions were interpreted as intended and were understood. All secondary school children appeared comfortable completing this type of self-completion questionnaire, perhaps because they are regularly asked to complete this style of questionnaire at school. 

Children in this age group understood the meaning of the food and drinks as intended. As with the primary school children though, “packets of snacks” tended to be interpreted as sweet foods. It was apparent that examples were required to illustrate some of the types of foods.

Following the cognitive interviewing, some changes were made to the wording of the questions, but the main changes were made to the delivery of the FPM. It was decided that the problems that primary school children had completing the SCQ meant it would work better if all questions were incorporated into the computer-assisted personal interview (CAPI). Primary school children would not be asked to complete the SCQ. 

Some routing problems were also observed with the secondary school children, mainly due to children not reading or not following the instructions. To try and limit this problem, some of the questions were incorporated into the CAPI which meant the interviewer could then score out some questions in the SCQ, based on the CAPI answers given, to prevent children mistakenly answering some questions in the SCQ. For example, if children said they never bought food at lunchtime, the interviewer could score out the questions relating to purchases made at lunchtime in the SCQ. The routing instructions that remained in the SCQ were highlighted more clearly in the final version of the questionnaire.

The questions included in the final CAPI and SCQ are outlined in Table 1 for younger and older children.

## 3. Results and Discussion

The main dietary survey aimed to achieve a representative sample of 1500 children aged 3–16 years. It was designed to be representative of children living in Scotland in terms of sex, ethnicity, urban-rural distribution, and age distribution. In the main dietary survey, 1906 children completed the interview. Further details about sampling are available in the published reports [13, 16]. Fieldwork for the FPM was conducted between June and November 2010. When interviews were conducted during the school holidays, parents and children were asked to recall information based on their previous school term.

All eligible primary school children from the dietary survey completed the FPM (*n* = 564). Of the eligible secondary school children (*n* = 653), 615 (94%) completed the SCQ questionnaire. There were no significant differences in response rates to the SCQ by sex or levels of socioeconomic deprivation. 

Despite making changes based on the findings from the cognitive testing phase, there were several errors in the SCQ data because of navigation and routing problems. When such errors occurred, children were excluded from the analysis as it was impossible to know which responses were correct. For example, if a respondent answered that they did not purchase food or drinks on the way home from school but then answered the questions about the type of food and drinks purchased at that time point, they were deemed as *not *purchasing food and drinks on the way home from school and were excluded from the analysis of food and drink purchasing at this time point. 

**Figure 1:**
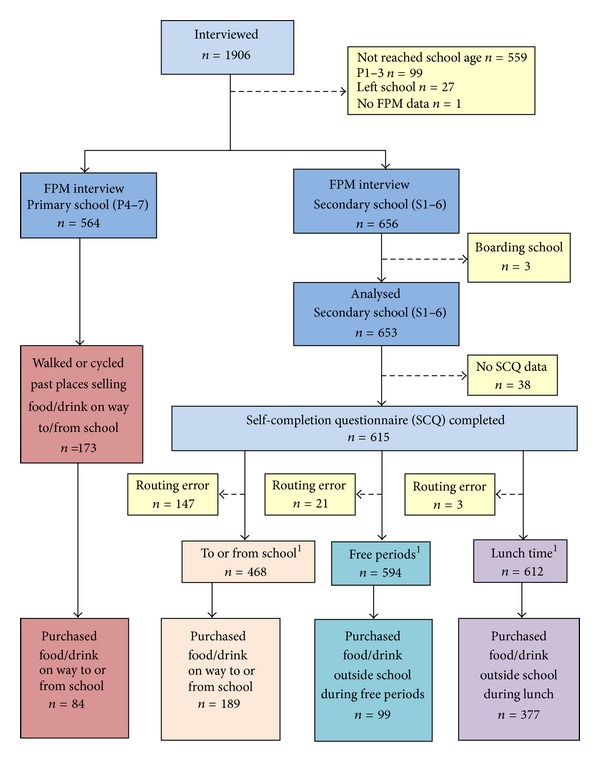
Sample available for the FPM data analysis. ^1^Represents children who reported opportunities to purchase food or drink.

Decisions about such navigation and routing errors had implications for the numbers available for the subsequent analysis, as indicated by Figure 1.

This is the first time a survey has been developed on food purchasing behaviour beyond the school gate using a nationally representative sample of children in Scotland. The results from this survey provide an overview of the food and drink purchasing habits of school children in Scotland and an insight into the issue of school children purchasing food and drinks high in fat and/or sugar on the way to and from school and in the vicinity of the school grounds at break times and lunchtime. 

Designing a module on a new topic presents several challenges. As the overall prevalence of food and drink purchasing outside of school was unknown at the time the study was designed, it was not possible to estimate in advance what size of sample would be required to provide sufficient numbers of children making purchases who could be asked detailed follow-up questions. As a consequence, a limitation of the results from the new module was the available sample size for exploring differences between subgroups (e.g., by age or deprivation quintile) and for exploring the influences on children's purchasing behaviour. The FPM data can now provide an estimate of the sample size required to ask more detailed follow-up questions and make comparisons between subgroups in future surveys.

Importantly, developing the FPM and reporting on the process of this development provides much needed information about children as survey respondents. It is crucial that children are given the opportunity to report on their own lives as information provided by proxy, from their parents, for example, does not usually result in data that is as accurate [17].

The combined use of the CAPI and SCQ data collection methods for secondary school children sometimes led to inconsistencies between the answers given in each mode, which meant that not all of the data could be used in the analysis. Some of these inconsistencies may have resulted from children choosing not to accurately disclose their purchasing habits in the presence of a parent or guardian during the CAPI; only 46% of secondary school children reported in the SCQ that they sometimes or always told their parents what foods or drinks they purchased at lunchtime, perhaps indicating that a significant proportion did not routinely discuss their food choices with parents and therefore might not be comfortable in doing so. Inconsistencies between the CAPI and SCQ may also have stemmed from a social desirability effect (e.g., wanting to answer all the questions to please the interviewer or their parents) as well as some children getting confused by the SCQ routing instructions. The decision about which questions to include in the CAPI was largely driven by a judgment about which were the least potentially sensitive topics. In many cases, it was also driven by the complex routing of questions; CAPI methods are preferable to self-completion methods in such cases, as routing errors are eliminated in CAPI as the computer programme automatically routes respondents to the next appropriate question, based on their previous responses. Complex questions can be difficult to answer for any respondent, not just children, and it was appropriate to ask children themselves about their own purchasing behaviours, rather than their parents [17], although this was not achieved to the same extent with regard to primary school children who were not given the option of a SCQ following the lessons learnt during the cognitive testing phase. Given that interviewers asked the parents or guardians of children under 12 questions in the CAPI, rather than asking the child directly, this may have also influenced the accuracy of the data collected. 

Despite these limitations, the survey provides timely and unique information about this important topic and has generated some useful methodological insights for future studies. For example, a computer-assisted self-completion interview (CASI) in which the children read questions on screen and input their own answers (as opposed to the CAPI which is interviewer administered) would be an alternative to the paper-based self-completion questionnaire and would help avoid future errors due to confusion with routing and overcome the issue of answering sensitive questions in front of parents or guardians.

CASI methods were not appropriate for this study because it would have added to the interview length (the paper-based SCQ was completed by the children while the adult continued with the CAPI). Whilst CASI methods are ideal for older children, an audio-CASI could also be developed for use with younger children or those with literacy problems. This would involve children listening to questions via headphones (rather than reading text on screen) and inputting their answers directly onto a computer or tablet. 

The survey format provided an appropriate method for exploring this topic, but a number of questions were raised which warrant further investigation. Using qualitative methodologies to explore food and drink purchasing beyond the school gate would facilitate an in depth understanding of some of the factors influencing children's purchasing behaviour. Such an approach would help uncover and explain the complexity of children's food and drink purchasing practices including exploring possible contradictions in their behaviour. The reasons children gave for leaving the school grounds at lunchtime and for purchasing/not purchasing food and drink were based on the list of reasons developed by the research team, which drew on their expertise and also on the relevant literature in this area. Qualitative work would allow children to voice their own reasons for purchasing food and drinks. The FPM module recorded the types of outlet which were most often used by children during the school day, but the survey could not explore in-depth *why *some outlets were used and not others, despite opportunities to purchase at a range of outlets. Qualitative research would help answer these questions and facilitate further exploration of the way that food and drink purchasing beyond the school gate might be contributing to children's intakes of NMES and SFAs.

## 4. Conclusion 

Assessing the availability and purchasing of energy dense foods by children from outlets in the vicinity of schools, termed “beyond the school gate,” is an important policy goal in Scotland and has relevance to other countries where obesity is a problem. The FPM was developed to address this issue. A number of methodological issues were raised during the development of the survey module, including that younger children experience problems using a self-completion questionnaire and that older children do not always successfully navigate a SCQ. The need to respect children's rights not to disclose their food and drink purchasing habits in front of parents is an important consideration and should inform the way that future surveys are developed. The FPM can be developed further, alongside qualitative research to look in more depth at the issues covered by the survey.

## Figures and Tables

**Table 1:** Questions included in the FPM.

Questions included	CAPI (primary school child aged 8–11 y^a^)	CAPI (secondary school child aged 11^a^–16 y)	SCQ (secondary school child aged 11^a^–16 y)
How the child travels to/from school	*✓*	*✓*	
Where the child eats, if at all, before school	*✓*	*✓*	
What places that sell food or drink the child walks or cycles past on way to/from school (opportunities to purchase)	*✓*	*✓*	
Who the child is with when walking or cycling to/from school	*✓*	*✓*	
Does the child purchase food or drinks on way to/from school	*✓*	*✓*	
Why the child never purchases food or drinks on the way to/from school	*✓*	*✓*	
Why the child purchases food or drinks on the way to/from school	*✓*		*✓*
Where the child buys food on way to/from school	*✓*	*✓*	
Frequency of purchasing certain food and drinks on way to/from school^a^	*✓*		*✓*
Parental influence^b^ on purchasing decisions on way to/from school, during break time/free periods, and at lunch time			*✓*
Places that the child can get to that sell food or drinks during free periods and lunch time		*✓*	
School rules about leaving school grounds during break times/free periods and at lunch time		*✓*	
Frequency of purchasing lunch provided by the school	*✓*		*✓*
Whether the child has a free school meal^c^	*✓*		*✓*
How long the child gets for lunch on a school day (in minutes)		*✓*	
How the child decides what to do at lunchtime		*✓*	
Whether the child purchases food or drinks during break time/free periods or at lunch time			*✓*
Reasons the child never buys food or drinks outside school during break time/free periods or at lunch time			*✓*
Reasons why the child leaves school grounds to purchase food or drinks during break time/free periods or at lunch time			*✓*
Where the child usually buys food or drinks outside school during break time/free periods or at lunch time			*✓*
Where the child most often buys food or drinks at lunch time outside of school and reasons why he shops there			*✓*
Frequency of purchasing certain food and drinks^b^ during break time/free periods or at lunch time			*✓*

^a^The age bands overlap as some children aged 11 years are still at primary school and some have started secondary school.

^b^The foods and drinks children were asked about were based on those included in the Food Frequency Questionnaire in the main dietary survey, supplemented with foods thought that might be purchased by children, like sandwiches. The list of food and drinks from the FPM can be found in the Appendix.

^c^Questions included whether the child tells their parent/guardian what food or drinks they buy and whether parents tell child what to buy/what not to buy on way to/from school, during break time/free periods, or at lunchtime.

^d^Children in the UK whose parents are on a low income are eligible for a free meal in school at lunchtime.

## Appendix

Children were asked how frequently they purchased the following food and drinks.

Hot or cold sandwiches, filled rolls, or baguettes.
Pizza, chips, pies, sausage rolls, or burgers.
Cereal bars, biscuits, cakes, including Danish pastries, doughnuts, or iced buns.
Crisps.
Sweets or chocolate.
Ice cream or ice lollies.
Pure fruit juice or smoothies.
Diet drinks, for example, Diet Coke, Ribena Light, or flavoured water.
Nondiet drinks, for example, Coke, Fruit Shoots, Ribena, Lucozade, Red Bull.
Plain water, including still and sparkling.
Plain or flavoured milk.
Tea, coffee, or hot chocolate.
Other food or drink (please tell us).
